# Rapid and sustained contact tracing training for COVID-19 in San Francisco: a training model for developing an emergency public health workforce

**DOI:** 10.3389/fpubh.2023.1125927

**Published:** 2023-06-30

**Authors:** Shayanne Martin, Anika Kalra, Alisa Jenny, Andrew D. Maher, Allison Foreman, Alejandro Chavez, Jayne Gagliano, Michael J. A. Reid, Debbie Bain Brickley

**Affiliations:** ^1^Institute for Global Health Sciences, University of California San Francisco, San Francisco, CA, United States; ^2^School of Medicine, University of California San Diego, San Diego, CA, United States; ^3^School of Medicine, University of California San Francisco, San Francisco, CA, United States; ^4^Department of Public Health, COVID-19 Command Center, San Francisco, CA, United States; ^5^Department of Medicine, University of California San Francisco, San Francisco, CA, United States; ^6^Department of Epidemiology and Biostatistics, University of California San Francisco, San Francisco, CA, United States

**Keywords:** public health training, COVID-19, contact tracing, curriculum development, training evaluation, participatory learning, outcome-based education, remote learning

## Abstract

The City and County of San Francisco was the first municipality in the United States to institute a COVID-19 contact tracing program. The San Francisco Department of Public Health (SFDPH) and the University of California, San Francisco (UCSF) created an outcome-based fully remote contact tracing curriculum using participatory learning methods to train non-public health emergency workers as contact tracers. Between April and December 2020, we trained over 300 individuals in contact tracing skills and procedures over three training phases. Using iterative curriculum design and Kirkpatrick’s evaluation methodology, we aimed to ensure high quality and successful person-centered contact tracing. The resulting curriculum consisted of 24 learning outcomes taught with six participatory skills development activities, asynchronous materials, and one-on-one contact tracer support. We collected more than 700 responses from trainees using various evaluation tools across the training phases, and contact tracers interviewed more than 24,000 contacts after training in our program. Our evaluations showed that knowledge and skills improved for most trainees and demonstrated the utility of the training program in preparing trainees to perform person-centered contact tracing in San Francisco. Local health jurisdictions and state health agencies can use this model of curriculum development and evaluation to rapidly train a non-public health workforce to respond to future public health emergencies.

## 1. Introduction

Contact tracing is a routine infectious disease control strategy used in the United States since the early twentieth century, most notably to slow the spread of syphilis, gonorrhea, tuberculosis, and HIV (1). To reduce disease transmission, public health workers identify and notify people who have been in close contact with an infected person and support them to follow behaviors to reduce the risk of infection through continuous exposure and reduce onward transmission (2). However, longstanding disinvestment in public health, staff shortages, and the rapid spread of COVID-19 meant that health departments across the U.S. struggled to expand contact tracing in time to contain COVID-19 at the start of the pandemic (3).

In January 2020, the first case of COVID-19 was detected in the United States, and it began spreading across the country. In March 2020, the City and County of San Francisco was the first municipality in the country to institute a community contact tracing program for COVID-19 (4, 5). The San Francisco Department of Public Health (SFDPH) partnered with the University of California, San Francisco (UCSF) to implement a remote program to train staff – many with no previous experience in public health – to be person-centered contact tracers (6).

Between April and December 2020, collaborators from UCSF and SFDPH trained 338 individuals in contact tracing, including furloughed civil servants, community health workers, and students. We created an outcome-based, participatory training curriculum and used the Kirkpatrick evaluation methodology (7) to ensure the workforce was prepared for person-centered contact tracing. This paper details our iterative curriculum development and subsequent learning evaluation strategy, both of which can be used as frameworks for health jurisdictions to rapidly train a lay workforce to respond to future public health emergencies.

## 2. Pedagogical framework

The training team designed the curriculum and evaluation in three phases, which we aligned to the progression of the pandemic in San Francisco: (1) Pilot, (2) Scale-Up, and (3) Capacity Strengthening. The training development process was iterative, with updates aimed to improve the learning experience and increase workforce performance, productivity, and self-efficacy. The curriculum required routine updates based on the emerging science of COVID-19, changes to San Francisco’s public health guidance, feedback received from supervisors on development needs of the workforce, and evaluation results of each training cohort. As the workforce expanded over time to include different professional backgrounds and experiences, this responsive training methodology helped meet the learning needs of the workforce.

After many iterations in training methodology, we created a final outcome-based curriculum that utilized participatory learning methods. We used this approach to ensure trainees could perform essential skills needed for contact tracing. In outcome-based education, the desired learner practices inform the curriculum content, organization, strategies, and assessment (8). We grouped the contact tracing outcomes by learning objectives, which we developed using the Revised Bloom’s Taxonomy Model (9). We prioritized participatory learning methods to maximize engagement in the remote training setting and provide practice opportunities to support contact tracers to confidently engage with COVID-19 close contacts. Participatory learning “encourages learning by doing things, using small groups, concrete materials, open questioning and peer teaching,” (10) all of which were used in the curriculum.

To objectively evaluate the program on a continuous basis, the training team utilized the Kirkpatrick Framework for Evaluation to measure learning outcomes (11). Widely applied to evaluate the results of training programs, the Kirkpatrick Framework consists of four levels of evaluation: (1) reaction, or trainee’s initial and immediate response to training; (2) learning, or the degree to which trainees meet learning outcomes of the training with knowledge and confidence gained; (3) behavior, or the degree to which trainees change their behavior as a result of knowledge and confidence gained; and (4) results, or the overall success of the training program measured with long-term outcomes. We used Levels 1–3 to evaluate the contact tracing training curriculum. Due to the difficulty of measuring the direct impact of contact tracing on COVID-19 rates, Kirkpatrick level 4 (results) was outside the scope of this evaluation.

Over the course of the San Francisco contact tracing program, the training team regularly revised the evaluation tools to align with updates to the curriculum and workforce performance indicators. Evaluations also assisted supervisors and the training team by identifying trainees who needed additional support prior to starting contact tracing.

## 3. Learning environment and pedagogical format

### 3.1. Training setting and team

The training team formed during the Pilot phase with three staff from UCSF’s Institute for Global Health Sciences (SM, DB, AM) who had prior experience in developing public health trainings. Our team grew over time to include up to 10 team members, including one member from SFDPH (JG), and was led by a training director (AJ) and a training manager (AK) from UCSF. All training occurred virtually using Zoom^™^ video conferencing (12) to adhere to San Francisco’s “shelter-in-place” public health ordinance. We utilized Zoom^™^ interactive features, including screen sharing, polling, breakout rooms, and participants’ video sharing. In the first two phases, we used email to disseminate training materials, communicate training schedules, and send evaluation tools to trainees. In the Capacity Strengthening phase, we used Microsoft Teams (13) to disseminate training materials and evaluation tools and email to communicate training schedules.

### 3.2. Training population

Our trainees were primarily civil servants recruited through the City and County of San Francisco Disaster Service Worker Program (Table 1) (4). Most trainees (64%) had no prior public health experience.

**Table 1:** Characteristics of San Francisco COVID-19 contact tracing trainees during three phases of training, April to December 2020^a^.

Training phase	Pilot	Scale-Up	Capacity Strengthening
Number of Trainees	37	108	193
*Types of workers*			
UCSF staff	37 (100%)	-	6 (3%)
SFDPH	–	–	5 (3%)
Student^b^	–	25 (23%)	48 (25%)
Disaster Service Worker	–	83 (77%)	78 (40%)
Redirected state worker	–	–	28 (15%)
CBO	–	–	28 (15%)^d^
*Languages spoken, in addition to English*			
Spanish	11 (30%)	19 (18%)	36 (19%)
Cantonese	–	–	11 (6%)
Mandarin	–	–	6 (3%)
Tagalog	–	–	4 (2%)
Other^c^	1 (3%)	–	12 (6%)

a Data recorded on training rosters.

b From City College of San Francisco, University of San Francisco, or University of California, San Francisco.

c Other languages spoken include Portuguese, Czech, Vietnamese, Malay, Japanese, Punjabi, French, Bulgarian, Hebrew, Arabic, Russian, Hindi, Urdu.

d Due to rounding, total adds to over 100%.

In the Pilot phase, we trained 37 UCSF Institute for Global Health Sciences staff. During the Scale-Up phase, we trained 83 Disaster Service Workers and 25 students, most of whom had limited or no public health experience. The largest group of Disaster Service Workers in our program were public library trainees; others included city attorney investigators, tax assessors, and building inspectors. The student trainees included UCSF medical and pharmacy students and health professions education students from UCSF and City College of San Francisco. During the final Capacity Strengthening phase, we trained an additional 78 Disaster Service Workers, 27 students, and 11 UCSF and SFDPH employees, as well as 28 redirected California state workers and 28 staff from community-based organizations (CBOs). Similar to the San Francisco Disaster Service Workers, most redirected California state workers had minimal public health experience. The city enlisted trainees from CBOs to help reach its diverse and vulnerable populations. While many of these trainees had no formal training in public health, CBO staff were more familiar with training concepts such as person-centered practice, motivational interviewing, and trauma-informed care. They also provided language concordance by speaking more of the primary languages spoken by San Francisco’s diverse population, which was associated with a higher likelihood of COVID-19 testing and referral to support services (14).

### 3.3. Curriculum and evaluation design and implementation

**Figure 1:**
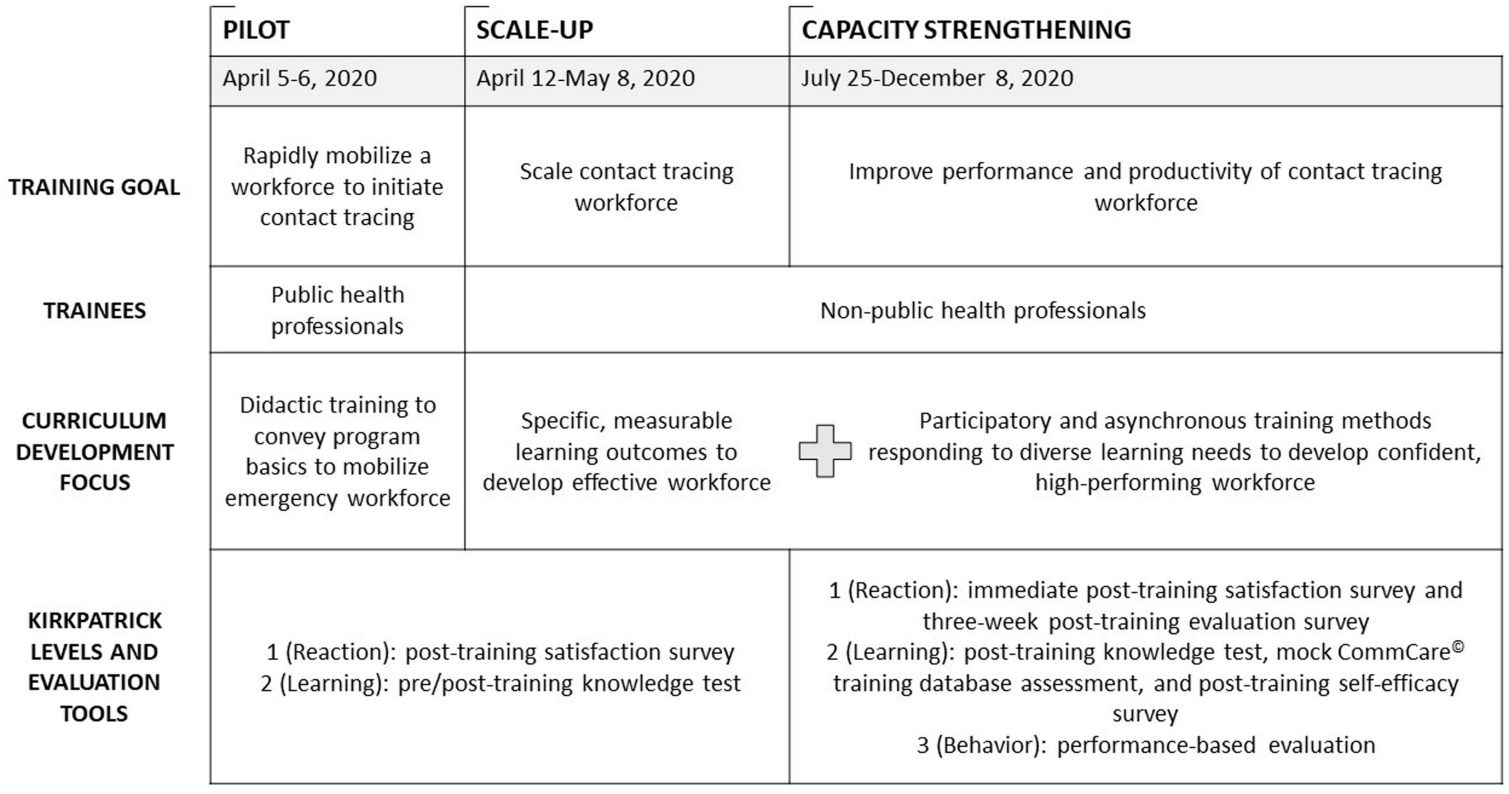
Evolution of curriculum and evaluation methodology during three phases of training for San Francisco COVID-19 contact tracers, April to December 2020.

The training team adapted the curriculum and evaluation methodologies as the contact tracing program evolved from Pilot to Scale-Up to Capacity Strengthening phases (Figure 1). Our training focused only on contact tracing; case investigation was completed separately by a SFDPH team during the period described in this paper.

#### 3.3.1 Pilot curriculum

By April 1, 2020, San Francisco had 625 cumulative cases of COVID-19, with a 7-day rolling average of 35 new cases a day (15). Pilot training took place on April 5–6, 2020 with the purpose of creating a capable workforce in response to the urgency to contact trace for COVID-19 (4). We developed training materials concurrently with the development of contact tracing procedures, and we piloted the system with the city’s first recorded COVID-19 contacts. We developed the Pilot curriculum in three days and delivered it as a three-hour training over two days to 37 UCSF global health staff. Content was limited to the history, definition, and processes of contact tracing, COVID-19 epidemiology, transmission and prevention, and use of digital tools for contact tracing, including a softphone application (RingCentral^™^ (16)) and a database application (CommCare^©^ by Dimagi (17)). Training methods consisted mostly of PowerPoint (18) presentations that adapted didactic content from existing contact tracing training tools for TB and HIV partner testing and included step-by-step screen captures of the softphone and database applications. Training concluded with one hour of role play interview practice. The same day that training concluded, UCSF contact tracers started making contact tracing calls independently.

#### 3.3.2. Scale-Up curriculum

SFDPH and UCSF health experts modelled COVID-19 rates and estimated that we needed 100–150 contact tracers for San Francisco’s population of 900,000 people (4). To meet this need, our training goal shifted to rapidly scale the contract tracing workforce by effectively training Disaster Service Workers from April 12 to May 8, 2020. In the six days between Pilot and Scale-Up phases, our collaborators developed standard operating procedures for contact tracing. In turn, the training team established learning objectives and outcomes that reflected the new procedures. We developed 24 specific, measurable learning outcomes (Table 2). We added activities to develop skills in motivational interviewing and calculating quarantine and isolation periods. Furthermore, trainees began shadowing existing UCSF contact tracers on one to two calls before making contact tracing calls independently.

**Table 2:** Learning objectives, learning outcomes, and skills development activities of Capacity Strengthening phase curriculum for San Francisco COVID-19 contact tracers, July to December 2020^a^.

Learning objective	Learning outcomes	Skills development activities
Explain basic COVID-19 epidemiology and containment strategies to COVID-19 close contacts	Define basic concepts of COVID-19 epidemiology Explain isolation and quarantine windows Understand the importance of wrap around services for the containment of COVID-19 Apply reference material (e.g., Standard Operating Procedure, FAQ) to supplement questions and guidance in the contract tracing interview	(A) Standard operating procedure scavenger hunt (in groups of 8–10 trainees, aided by facilitator) (B) Small group Microsoft Teams scavenger hunt (in groups of 3, aided by a roaming facilitator) (C) Contact tracing role play, including data entry into mock contact tracing database (in pairs, data entry “graded” by training team post-practice)
Relate standardized policies about isolation, quarantine, and testing to the needs of COVID-19 close contacts	Evaluate symptoms for infection risk Determine whether the contact needs to quarantine or isolate Recall standardized recommendations and policies about isolation, quarantine, and testing	(A) Standard operating procedure scavenger hunt (in groups of 8–10 trainees, aided by facilitator) (C) Contact tracing role play, including data entry into mock contact tracing database (in pairs, data entry “graded” by training team post-practice)
Use best practices of maintaining confidentiality while undertaking contracting work	Obtain consent to conduct contact tracing interview Protect the confidentiality of cases and contacts	(C) Contact tracing role play, including data entry into mock contact tracing database (in pairs, data entry “graded” by training team post-practice) (D) Practice making calls and sending SMS messages with softphone application (in groups of 3, aided by a roaming facilitator)
Employ person-centered communication strategies to support COVID-19 close contacts to isolate or quarantine	Apply motivational interviewing and health coaching techniques Demonstrate cultural humility Apply best practices for using an interpreter with language discordant contacts	(C) Contact tracing role play, including data entry into mock contact tracing database (in pairs, data entry “graded” by training team post-practice)
Determine the physical and psychosocial needs of COVID-19 close contacts in order to safely isolate or quarantine	Assess home/living environment Measure need for isolation hotel Measure need for delivery of food, masks, and cleaning supplies Measure need for additional resources, such as pet care needs or pharmacy pick-up	(A) Standard operating procedure scavenger hunt (in groups of 8–10 trainees, aided by facilitator) (C) Contact tracing role play, including data entry into mock contact tracing database (in pairs, data entry “graded” by training team post-practice) (E) Practice making food and cleaning supplies referrals to the Isolation & Quarantine Unit (in groups of 3, aided by a roaming facilitator)
Facilitate referral to healthcare services and resource care coordination for COVID-19 close contacts	Analyze medical history to determine risk for critical infection and need for clinical consult Make food referrals *via* city services for food-insecure populations Make isolation hotel referrals for eligible populations Document testing referrals Make testing appointments for contacts	(A) Standard operating procedure scavenger hunt (in groups of 8–10 trainees, aided by facilitator) (C) Contact tracing role play, including data entry into mock contact tracing database (in pairs, data entry “graded” by training team post-practice) (E) Practice making food and cleaning supplies referrals to the Isolation & Quarantine Unit (in groups of 3, aided by a roaming facilitator) (F) Practice viewing, searching, and entering data in contact tracing database (individual practice facilitated by trainer in large group)
Use software applications to complete contact tracing	Manipulate script to conduct a comprehensive interview Accurately document contact tracing interview and outcomes (e.g., referrals, guidance given) in contact tracing database Use softphone application to conduct contact tracing interview, including how to do warm transfers	(C) Contact tracing role play, including data entry into mock contact tracing database (in pairs, data entry “graded” by training team post-practice) (D) Practice making calls and sending SMS messages with softphone application (in groups of 3, aided by a roaming facilitator) (F) Practice viewing, searching, and entering data in contact tracing database (individual practice facilitated by trainer in large group)

a Objectives, outcomes, and skills development activities evolved over the course of curriculum iteration. For simplicity, this table shows the final objectives, outcomes, and skills development activities.

As the workforce grew, two challenges appeared. First, digital literacy, which was critical in a fully remote work environment, varied among contact tracers. Contact tracers needed to navigate between several different software applications and websites while making calls. Many reported IT problems or felt overwhelmed performing simultaneous tasks online. Second, those trained during Scale-Up required substantial on-the-job support from Pilot phase contact tracers, including shadowing and help performing procedures. Supervisors requested the training team include more practice opportunities during training to reduce the on-the-job training burden.

#### 3.3.3. Capacity Strengthening curriculum

In the Capacity Strengthening phase (July 25, 2020–December 8, 2020), we expanded the training curriculum to include virtual IT office hours via Zoom^™^, more skills development activities, and an onboarding process (Table 2). We introduced IT office hours to support staff who required additional help installing or developing proficiency with digital contact tracing tools. Skills development activities included participatory methods such as a small group standard operating procedure scavenger hunt and practice interviews using a mock training database with fictitious cases and contacts. We provided activity instructions both in writing and by facilitators in Zoom^™^ breakout rooms. The onboarding process consisted of: (1) one group shadow shift, where 8–10 trainees shadowed an experienced tracer for a four-hour contact tracing shift to listen to a variety of contact tracing encounters and observe data entry via a live screen shared in Zoom^™^; (2) one reverse-shadow shift, where an experienced tracer shadowed one trainee for a four-hour contact tracing shift with on-the-call support and post-call feedback; and (3) three buddy shifts, during which a new tracer was paired with an experienced tracer to provide one-on-one support and answers to questions about procedures via instant messaging on the softphone application, RingCentral^™^, or video calling on Zoom^™^. We obtained verbal consent for shadowing and reverse-shadowing for the purpose of training from all contacts, and experienced contact tracers volunteered to be shadowed.

During this phase, the training team also added asynchronous training (i.e., independent and self-paced) to provide a consistent yet flexible approach to retraining the existing workforce on updated guidelines. Self-study materials included video tutorials and one-page reference sheets that presented newer concepts and skills (e.g. cultural humility and health equity), updates in contact tracing standard operating procedures, and additional areas of concern expressed by the workforce. For example, contact tracers requested more information on how to use interpretation services to communicate with contacts who spoke a different language. In response, we provided PowerPoint slides and video demonstrations teaching the principles and practices for using an interpreter. We also introduced trainings and training materials in Spanish to increase accessibility to Spanish-speaking contract tracers with limited English proficiency.

#### 3.3.4. Pilot evaluation

Prior to the Pilot phase, the training team created a pre-/post-training survey in Qualtrics (19) to measure Kirkpatrick Levels 1 (reaction) and 2 (learning). We included five open-ended reaction questions in the post-training survey which asked trainees to assess their preparedness for contact tracing and suggest ways to improve the training for non-public health trainees in the next training phase. To measure learning, we included nine knowledge questions in both the pre-and post-training survey on contact tracing procedures and application in hypothetical scenarios to measure trainees’ preparedness for contact tracing. No passing score was set. Additionally, one Likert scale question measured self-efficacy and preparedness for contact tracing.

#### 3.3.5. Scale-Up evaluation

In preparation for scaling the workforce, the training team updated evaluation tools for Levels 1 (reaction) and 2 (learning) to match the new learning objectives and outcomes. We reduced reaction questions to two or three open-ended questions and one Likert scale question, depending on the intra-phase training cohort, to assess satisfaction with training and request feedback on training, respectively. A knowledge test contained nine, five, or 26 questions, depending on the training cohort, to assess mastery of the learning objectives. No passing score was set. We included the same Likert scale question to measure self-efficacy and preparedness.

#### 3.3.6. Capacity Strengthening evaluation

To respond to requests from supervisors to strengthen capacity of contact tracers, we revised the evaluation strategy to include additional tools (Figure 1) (all tools described for this phase are in the Supplementary Material).

We measured Level 1 (reaction) with two tools at different timepoints. First, we distributed a post-training satisfaction survey via Zoom^™^ at the conclusion of the final training session. This survey consisted of ten multiple choice and Likert scale questions and three open-ended questions that asked respondents to evaluate the benefits and effectiveness of training. Second, we implemented a training evaluation three weeks after the completion of training to measure the perceived usefulness and relevance of the training curriculum once trainees started contact tracing. This survey was adapted from an evaluation tool used by California’s COVID-19 Virtual Training Academy (20, 21), which was a statewide training initiative developed after the initiation of the San Francisco contact tracing program. It included eight Likert scale questions and space for additional comments.

We measured Level 2 (learning) with three tools. First, we implemented a post-training knowledge test consisted of 13 questions that measured contact tracing learning outcomes. This contained eight questions related to learning objectives on isolation and quarantine guidance. The remaining five questions related to understanding CommCare^©^, Microsoft Teams, and Amazon Connect (22), a softphone application adopted by SFDPH during this phase. By programming an advanced feature in Qualtrics, we allowed multiple attempts until trainees achieved a passing score of 70%. Second, we built a mock CommCare^©^ database filled with hundreds of fictional cases and contacts to enable a database assessment in response to a simulated contact tracing encounter. In the mock database, we asked trainees to record collected data and the correct public health actions to take (e.g. schedule COVID-19 test) based on the mock scenario. We coded 31 data points for correctness or completeness. Third, we iterated a post-training self-efficacy survey from the first two phases with seven Likert scale questions and implemented it one day after training to measure confidence and self-efficacy with contact tracing skills.

In Capacity Strengthening, we measured Level 3 (behavior) with a performance-based evaluation, consisting of a live contact tracing interview evaluated by an experienced contact tracer shadowing the call. We evaluated trainees on overall interviewing skills and performance on learning outcomes using a standard rubric. Team supervisors and training team members reviewed the completed evaluations to categorize trainees as either ready for independent contact tracing or needing one-on-one support from training team members, supervisors, or peer support provided by experienced contact tracers.

## 4. Results

Our iterative curriculum development resulted in a final curriculum of six participatory skills development activities, reinforced by additional asynchronous methods and one-on-one support as needed, to support trainees’ achievement of the 24 learning outcomes (Table 2). Our training agenda with details of the curriculum and materials is available in the Supplementary Materials; further materials can be made available upon reasonable request to the corresponding author. Among the 338 trainees who participated in training, we received and analyzed 768 evaluation responses across all tools from the three training phases.

Indicators of programmatic reach showed that our contact tracers interviewed 24,790 contacts from April 1, 2020 - May 31, 2021 and referred more than 13,072 contacts to COVID-19 testing from April 13, 2020–May 30, 2021 (23). Early outcomes (Kirkpatrick Level 4) reported elsewhere indicate that we successfully notified 83.8% of close contacts, 37.6% of close contacts were tested for COVID-19, and 9.9% of close contacts were newly diagnosed with COVID-19 (24).

### 4.1. Evaluation results – level 1 (reaction)

We measured reaction during all three phases of the contact tracing program. During the Pilot phase, 57% of trainees (*n* = 21) participated in the post-training survey. Trainees thought training could be improved with increased time for Q&A, more hands-on CommCare^©^ practice, and interactive sessions with live demos. Additionally, trainees requested job aids and more information on testing availability and eligibility, isolation versus quarantine, and the rationale behind contact tracing. Trainees liked the clear presentations, felt the training was done well considering it was created in a short period of time, and reported information was delivered clearly, with well-suited multiple-choice questions for a non-public health audience.

In the Scale-Up phase, 87% of trainees (*n* = 94) participated in post-training surveys. Of those that participated, 45 trainees were asked about satisfaction; 85% were very satisfied and 15% were moderately satisfied with training. In qualitative feedback measuring reaction, trainees requested more information on how to handle difficult situations and questions from contacts. They also requested more training on the CommCare^©^ database and RingCentral^™^ softphone applications, including through additional mock practice, group breakout sessions, and shadowing experienced tracers, and also requested more access to training materials and supportive job-aids. Trainees stated that training was engaging and impressive. They reported that basic interview training along with reviewing the standard operating procedures, observing demonstrations, and reviewing practice scenarios was useful.

During the Capacity Strengthening phase, trainees rated the overall contact tracing training an average of 8.3 out of 10 (range of 4–10) on the post-training satisfaction survey with a 45% participation rate (*n* = 87). Additionally, 94% of respondents understood the goals and purpose of training and 92% said training activities were helpful for learning. The training evaluation survey had a 14% completion rate (*n* = 27) and showed that 92% of respondents felt proficient/comfortable in topic areas after training, 89% felt prepared for contact tracing after completion of training, and 80% found training activities effective. Contact tracers identified that script practice with contact tracing role play and practice making calls were the most useful for learning.

In the Capacity Strengthening phase, the qualitative feedback we gathered three weeks after training revealed that trainees liked the opportunity to practice with role play and in breakout groups, found interactive activities and live discussions helpful, and felt supported by trainers. Open ended responses also showed that although a lot of content was covered in training, contact tracers became more comfortable after being on the job for a few weeks. For example, one new tracer responded, “I was intimidated by the amount of new material to review for the program, but I quickly became comfortable as the program progressed, and I gained more experience.” In addition, contact tracers recognized efforts to gather feedback and adjust training in response. One contact tracer commented, “I love that you care about us by asking for our feedback and input and adjusting the training and programs accordingly. I feel valued and part of the team. Keep up with the surveys and feedback and work.”

### 4.2. Evaluation results – level 2 (learning)

**Figure 2:**
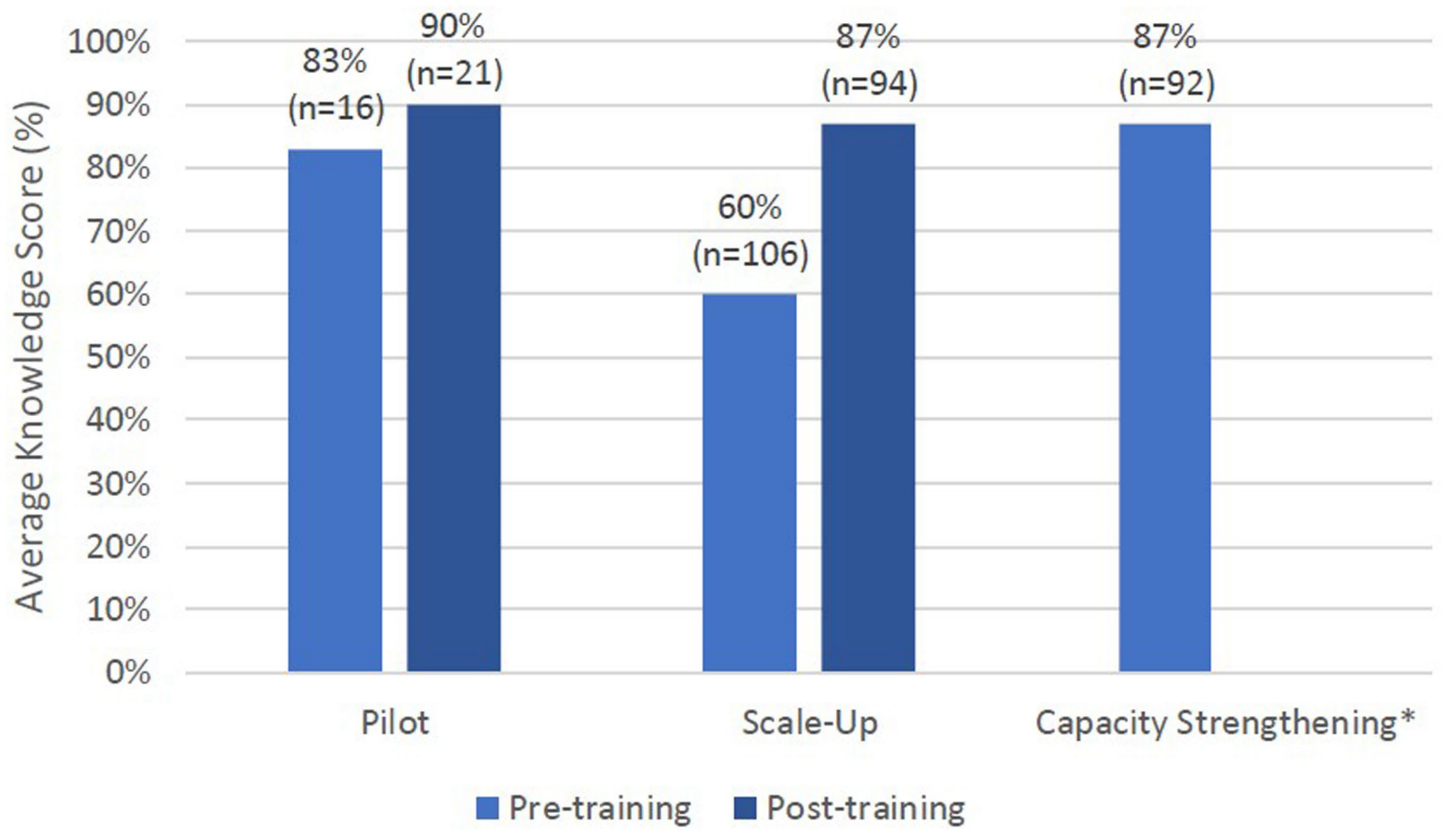
Average knowledge scores for all phases of training. Pilot phase test had 6 questions, Scale up phase test had 6, 5, or 26 knowledge questions, and Capacity Strengthening phase test had 13 questions. ^*^Calculated an average passing score for Capacity Strengthening phase with average of best attempt. Average of all attempts was 83%. Pre-training knowledge not measured in this phase.

In all phases of the contact tracing training program, we utilized a knowledge test to measure Learning (Figure 2). The Pilot knowledge test had variable participation rates from pre-training (43%, *n* = 16) to post-training (57%, *n* = 21) and showed an average improvement in scores by seven percentage points from 83 to 90%. During the Scale-Up phase, participation in the knowledge test varied from 98% (*n* = 106) pre-training to 87% (*n* = 94) post-training, and scores increased by an average of 17 percentage points from an average of 60% to 87%. During the Capacity Strengthening phase, 48% (*n* = 92) of trainees participated in the knowledge test and scored an average of 87% out of their best attempts; the average of all attempts was 83%. Additionally, the post-training knowledge test had a 90% overall pass rate (score > 70%). Of this group, 67% (*n* = 56) passed with one attempt, 27% (*n* = 22) passed in a second attempt, 5% (*n* = 4) passed with a third attempt, and 1% (*n* = 1) passed in their fifth attempt. The 10% of trainees who did not pass took the test no more than twice and started contact tracing regardless with extra support from staff and trainers, as the SFDPH mandate was to activate all reassigned workers to contact tracing at the end of training regardless of evaluation scores due to the emergency nature of the pandemic.

**Figure 3:**
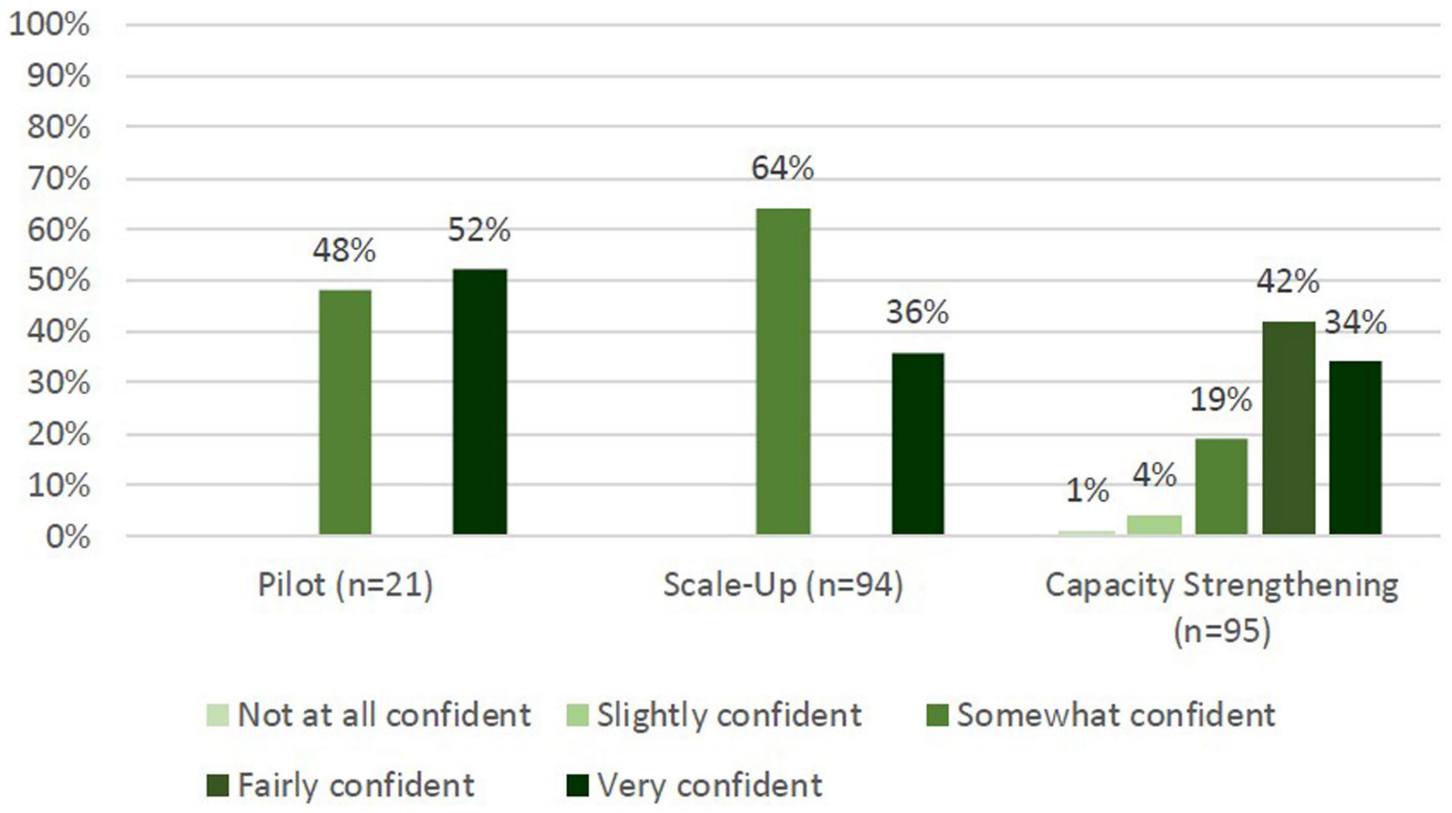
Post-training self-efficacy of trainees for contact tracing. Pilot and Scale-Up phases had 1 question with answer options: not at all confident, somewhat confident, and very confident. Capacity Strengthening had 7 questions and added answer options: slightly confident and fairly confident.

To measure self-efficacy in the Pilot phase, 57% (*n* = 21) of trainees participated in the evaluation. Of those, 52% (*n* = 11) felt “very confident” to begin contact tracing and the remaining 48% (*n* = 10) of trainees felt “somewhat confident.” No one responded with “not at all confident.” During the Scale-Up phase, 87% (*n* = 94) of trainees responded, of whom 36% (*n* = 34) felt “very confident” and the remaining 64% (*n* = 60) felt “somewhat confident” to begin contact tracing. No one responded with “not at all confident.” Finally, during Capacity Strengthening, 49% (*n* = 95) of trainees participated and on average across seven questions, 34% (*n* = 32) felt “very confident,” 42% (*n* = 40) felt “fairly confident” and 19% (*n* = 18) felt “somewhat confident” to begin contact tracing (Figure 3). Only 4% (*n* = 4) and 1% (*n* = 1) of trainees responded with “slightly confident” and “not at all confident” respectively.

We had a 41% (*n* = 80) participation rate on the CommCare^©^ database assessment in the Capacity Strengthening phase, and trainees achieved a 90% pass rate (score >= 70%) with an average score of 85% (range from 35% to 100%).

### 4.3. Evaluation results – level 3 (behavior)

Our performance-based evaluation, which had a participation rate of 61% (*n* = 118), had a 98% pass rate on the first attempt by trainees based on the rubric. In general, trainees completed all required parts of a contact tracing interview, with areas of improvement being improving the flow of the interview and using motivational interviewing techniques. Evaluators provided qualitative feedback that revealed many trainees felt nervous prior to making their first call but that they performed well and reported the experience built their confidence to begin contact tracing.

## 5. Discussion

This paper describes how the San Francisco Department of Public Health collaborated with a partner university, University of California, San Francisco, to rapidly initiate, adapt, and sustain a contact tracing training program during a public health emergency. Our training approach was noteworthy for its frequent iterations as we adopted new information and policies to contain the pandemic. We enhanced our approach from didactic to participatory to optimize skill building and improve self-efficacy. We made efforts to increase accessibility of the curriculum to Spanish-speaking trainees with limited English proficiency and increase training opportunities on technology for trainees starting from a place of lower digital literacy.

The Kirkpatrick Framework proved useful for evaluating training outcomes in real-time to monitor the contact tracing training program and contribute to quality improvement of the contact tracing workforce. Our evaluation results demonstrate the utility of the training program in preparing trainees to achieve the learning outcomes required to perform contact tracing in San Francisco. Most trainees demonstrated increases in knowledge, skills and self-efficacy. In the first two phases, we did not establish “passing scores” due to the urgency to deploy contact tracers. Rather, the training team monitored increases from pre- to post-training scores to approximate readiness for contact tracing and checked in with contact tracers with scores much lower than their peers. By the third phase, we were able to set a passing score to encourage additional self-study and retaking of tests even though we could not prevent a trainee from deploying to contact tracing work. We also had a direct line to supervisors to request on-the-job training and shadowing opportunities for trainees who did not pass. Additionally, we provided evaluation results to the SFDPH/UCSF Contact Tracing and Case Investigation Quality Assurance and Improvement team to inform performance monitoring.

Several health jurisdictions in the U.S. have published descriptions and outcomes of their contact tracing programs (24–27), but there is a scarcity of literature on training models and evaluations, particularly blended trainings used by specific local health jurisdictions. By comparing and assessing different models of training used during the COVID-19 pandemic, the public health community will be better positioned to optimize contact tracing efforts in the future. To the best of our knowledge, our paper is the second to report both a detailed description and evaluation of a replicable blended training model used by a local health jurisdiction in the United States (28).

The first, reported by Strelau et al. (28), was by the University of Pennsylvania in collaboration with the Philadelphia Department of Public Health. They trained 130 volunteers with a background in health, public health or social work for contact tracing from April 2020–May 2021. Common features between Philadelphia and San Francisco training programs include: teaching the science of COVID-19 and contact tracing, operational workflow and digital systems training, role play to practice contact tracing calls (including navigating difficult situations), and reference materials including frequently asked questions and contract tracing scripts. Both programs began with rapid development of emergency training and ended with increased reliance on asynchronous training and upskilling the existing workforce on trauma-informed approaches such as motivational interviewing and cultural humility. A key difference, however, is that the final San Francisco model blended synchronous and asynchronous training approaches, while the Philadelphia program transitioned to a fully asynchronous training (28).

Furthermore, when comparing evaluation methodologies, both the San Francisco and Philadelphia training program utilized pre- and post-tests to measure changes in trainee knowledge and self-efficacy (Kirkpatrick Level 2) (28). Unique to our evaluation methodology were the reaction surveys (Kirkpatrick Level 1) and assessment of contact tracing behavior (Kirkpatrick Level 3). We also focused on redesigning training based on test results and workforce learning needs. Our program instituted training evaluation from the very first emergency cohort, compared to the Philadelphia program, which began assessing trainees in their second cohort (28). Neither training program assessed trainees following asynchronous training, which is an area of improvement for the future. Additionally, outside of our program, we did not find evidence of any assessment involving a mock contact tracing interview complete with data entry and recorded public health action steps in a mock database. As a unique feature of our training program, we advocate for its utility as a comprehensive performance-based evaluation.

Our program can also be compared to state and national training programs to glean lessons learned. California’s COVID-19 Virtual Training Academy (20, 21) provided statewide contact tracing training and was developed after the initiation of the San Francisco contact tracing program with assistance from UCSF training team members. Results of their similar three-week post-training survey support the use of a delayed post-training tool to assess the effectiveness of preparing trainees (29). Furthermore, the Association of State and Territorial Health Officials (ASTHO), a national organization, made an asynchronous training freely available to build the COVID-19 response workforce across the United States (30) and conducted and published an evaluation of its training (31). On demand courses can be utilized by local jurisdictions as a starting point to efficiently teach an introduction to contact tracing, illustrated by that fact that in the first eight months of the ASTHO training, over 90,000 individuals completed the course (31). Of relevance, the San Francisco training program incorporated the ASTHO training as self-study material starting in the Capacity Strengthening phase (30). However, in addition to ASTHO, our program included localized training to teach the software systems required to conduct contact tracing and the jurisdiction-specific testing and support services available.

We suggest the San Francisco training model can be used by other local health agencies to rapidly train a lay workforce to respond to future public health emergencies. Specifically, we iterated our curriculum to be a combination of asynchronous and synchronous materials, which can be easily utilized in a remote environment. We used participatory learning methods, demonstrations, one-on-one support, shadowing shifts, and self-study materials to ensure the success of our contact tracers. We have included our training agenda with details of curriculum and materials in the Supplementary Materials; further materials can be made available upon reasonable request to the corresponding author. We have also included our final evaluation tools in the Supplementary Materials in the hopes that, in a future public health emergency, our work is replicable and can serve as a model for other local health jurisdictions across the nation. However, we would like to propose the following considerations and lessons learned for program implementers when developing a contact tracing training program during a disease outbreak: first, frequent changes to content are necessary in the setting of an evolving epidemic due to changes in available health and social resources, in public health guidelines, and utilization of new and changing technology; second, after the initial urgency of developing a basic contact tracing workforce, the curriculum focus should shift to differentiating learning methods to improve trainee satisfaction, learning, and behavior; third, program implementers should conduct a comprehensive evaluation strategy and closely monitor of results to respond to capacity, behavior, and confidence gaps in the workforce; fourth, contact tracing training must be jurisdiction-specific to maximize success, including training on software systems and technology utilized in the jurisdiction and social supports available for contacts and cases in the region.

### 5.1. Limitations

There were several constraints to the training program and evaluation. Many of the limitations were due to the need for rapid iteration and implementation of contact tracing. The training team had to constantly update the content and evaluation tools because of the rapidly changing understanding of COVID-19 and resulting changes in the SFDPH COVID-19 policies and procedures. Changes occurred frequently, including within training phases, leading to limitations in comparing evaluation results between phases. Trainings were often scheduled on short notice, at times less than one week in advance, which was challenging for both trainees and the training team. Due to the need for trainees to begin contact tracing immediately following the training, we had a short time period to remind trainees to complete evaluations and trainees had a short time period to reflect on materials and complete evaluation. This contributed to the low completion rate for most evaluation tools, which limited the understanding of the results of the training curriculum.

Challenges to training itself were related to the virtual training environment. Participants were often on Zoom^™^ for four or more hours at a time, resulting in Zoom^™^ fatigue by trainees and trainers. Furthermore, trainees relied on and greatly benefitted from experienced contact tracers and supervisors for shadowing and reverse-shadowing shifts, but there was low willingness among experienced tracers to provide these opportunities due to high workload. This resulted in a few experienced tracers carrying the majority of the time burden to help onboard new contact tracers.

Additionally, we believe the speed at which we iterated the curriculum and evaluation tools was foundational to developing a quality contact tracing workforce, but note the effort and time required to make frequent changes were significant and should be considered by program implementers choosing to take a similar approach. Nonetheless, we propose our approach to be a feasible model to rapidly train a lay workforce in response to future public health threats in local health jurisdictions.

Finally, a more robust evaluation strategy would have included tools to evaluate the asynchronous training methods and the results of the training program (Kirkpatrick Level 4).

### 5.2. Conclusion

Contact tracing remains a recommended tool for preventing the spread of COVID-19 in vulnerable populations and for other infectious disease outbreaks (32). Timely and effective responses to disease outbreaks and other public health emergencies require rapid mobilization of a capable workforce. When a skilled workforce does not already exist, one must be quickly developed. Our approach in San Francisco provides a blueprint for how to rapidly train a new public health workforce at the start of a public health emergency and then iterate training to improve the quality and person-centeredness of the workforce over time.

## Data availability statement

The raw data supporting the conclusions of this article will be made available by the authors, without undue reservation.

## Author contributions

MR conceptualized and led the contact tracing program. DB, AM, AJ, SM, and AK led curriculum pedagogy and design, with help from AF, AC, and JG on curriculum activities. SM, AK, AJ, AC, AF, AM, DB, and JG implemented training. SM and AK designed and implemented curriculum evaluation with assistance from AF. AC, AF, and JG assisted with evaluation data collection and analysis. SM conceived of the article’s design. AK led analysis and interpretation of data. SM and AK drafted the article, and revised it critically. DB, MR, and AJ made substantial contribution to the drafting of the article. AM made substantial contribution to the revision of the article. All authors contributed to the article and approved the submitted version.

## Funding

Training was supported by the San Francisco Department of Public Health (Contract ID # 1000017373).

## Conflict of interest

The authors declare that the research was conducted in the absence of any commercial or financial relationships that could be construed as a potential conflict of interest.

## Publisher’s note

The claims expressed in this article are solely those of the authors and do not necessarily represent those of their affiliated organizations, or those of the publisher, the editors and the reviewers. Any product that may be evaluated in this article, or claim that may be made by its manufacturer, is not guaranteed or endorsed by the publisher.
