# Supplementary material for: Rapid and sustained contact tracing training for COVID-19 in San Francisco: a training model for developing an emergency public health workforce
Source: Front Public Health. 2023 Jun 30;11:1125927. doi: 10.3389/fpubh.2023.1125927 (PMC10347383; doi:10.3389/fpubh.2023.1125927)
Supplement: Supplementary file 1 [file Table_1.docx]

***Supplementary Material***

**Rapid and sustained contact tracing training for COVID-19 in San Francisco: a training model for developing an emergency public health workforce**

Shayanne Martin, MPH^1†^, Anika Kalra, MS^1†*^, Alisa Jenny, MPH^1^, Andrew D Maher, MPH^1^, Allison Foreman^2^, Alejandro Chavez^3^, Jayne Gagliano^4^, Michael J A Reid, MD, MPH^1,3,5,6^, Debbie Bain Brickley, DrPH, MPH^1^

^1^Institute for Global Health Sciences, University of California San Francisco, San Francisco, CA, USA

^2^School of Medicine, University of California San Diego, San Diego, CA, USA

^3^School of Medicine, University of California San Francisco, San Francisco, CA, USA

^4^COVID-19 Command Center, Department of Public Health, San Francisco, CA, USA

^5^Department of Medicine, University of California San Francisco, San Francisco, CA, USA

^6^Department of Epidemiology and Biostatistics, University of California San Francisco, San Francisco, CA, USA

*** Correspondence:**Anika Kalra
anika.kalra@ucsf.edu

**^†^ Equal contribution and first authorship:** These authors contributed equally to this work and share first authorship

**1 Supplementary Evaluation Data**

| **Level 1. Post-training Satisfaction Survey** | |
| --- | --- |
| **How confident are you in your ability to conduct contact tracing calls?** | Very confident, somewhat confident, not confident |
| **I clearly understood the purpose and goals of the training.** | Strongly agree, agree, neither agree nor disagree, disagree, strongly disagree |
| **The activities of the technical training helped my learning.** | Strongly agree, agree, neither agree nor disagree, disagree, strongly disagree |
| **I was offered a variety of ways to learn the material during the training.** | Strongly agree, agree, neither agree nor disagree, disagree, strongly disagree |
| **The trainers were prepared and knowledgeable.** | Strongly agree, agree, neither agree nor disagree, disagree, strongly disagree |
| **The trainers offered constructive feedback.** | Strongly agree, agree, neither agree nor disagree, disagree, strongly disagree |
| **The email communications to trainees was clear and easy to follow (agenda, what to expect, and how to access meetings and materials).** | Strongly agree, agree, neither agree nor disagree, disagree, strongly disagree |
| **Please rate the usefulness of each activity to prepare you as a contact tracer:**   - SOP scavenger hunt - Isolation & quarantine unit services - Topic Presentations - Amazon Connect practice - CommCare Practice - Microsoft Teams presentation - Self-study - Contact tracing mock interview videos - Readings | Not at all useful, somewhat useless, neutral, somewhat useful, very useful |
| **How do you rate the Technical Training? (1= Very poor; 10= Exceeded expectations)** | 1-10 |
| **What aspects of the training did you enjoy the most?** | Free response |
| **What is the most important element you took away from this training?** | Free response |
| **Please share any suggestions you have to improve this technical training.** | Free response |

| **Level 1. Training Evaluation** | |
| --- | --- |
| **What is your opinion on the organization of the contact tracing program?**   - Contact Tracing training program - Contact Tracing SOP - Contact Tracing Script in CommCare - Shift Scheduling - Other support by workforce lead (your POC for scheduling) - In-briefs/Out-briefs with Team Leads - Assistance provided by Team Leads during shift | Needs improvement, meets expectations, exceeds expectations |
| **What is your opinion on the ease of use of these digital tools?**   - Amazon Connect - CommCare Application | Needs improvement, meets expectations, exceeds expectations |
| **Contact Tracing Interview Self-Assessment  - Please assess how proficient/comfortable you are in the following areas.**   - Explaining confidentiality - Assessment of symptoms and medical history - Assessment of COVID-19 exposure - Categorizing contact's risk - Providing guidance on self-isolation & quarantining - Referring contacts for testing - Referring contacts for social support services (e.g. food) - Completing the Person Under Investigation (PUI) - Using a translator | Extremely proficient/comfortable, very proficient/comfortable, moderately proficient/comfortable, slightly proficient/comfortable, not proficient/comfortable at all |
| **Please assess your Overall Job Perception and Preparedness**   - I feel prepared to do this work. - I feel as though I can do this work well. - I feel as though I am making a difference through this work. - I feel I am prepared to use motivational interviewing skills. - I feel I am prepared to use my health coaching skills. - I feel I am prepared to handle difficult situations. - I feel I am prepared to use Amazon Connect. - I feel I am prepared to use CommCare. | Strongly disagree, disagree, undecided, agree, strongly agree |
| **Do you feel you would have benefited from additional training on any of the following? (mark all that apply)**   - Epidemiology of COVID-19 - Comprehensive containment (Quarantine and Isolation) - Interviewing skills and building rapport - Health coaching - Addressing challenging situations - Using CommCare - Using AmazonConnect - Using a translator | Need more knowledge, need more practice |
| **What is your opinion on the effectiveness of these aspects of training?**   - Virtual Training Academy - Contact Tracing Training Curriculum (2 day Zoom training) - Training Shifts - Onboarding Shifts - Targeted follow up support: TL assistance - Targeted follow up support: Office Hours | Needs improvement, meets expectations, exceeds expectations, not applicable |
| **Additional comments?** | Free response |

| **Level 2. Post-training Knowledge Test*** | |
| --- | --- |
| **Which of the following most accurately describes the City of San Francisco (SF)’s guidance for isolation and quarantine of close contacts of persons with COVID-19?** | - *Persons with symptoms of COVID should stay home and isolate themselves from other people in the house for 10 days.* - Persons with no symptoms of COVID should stay home and isolate themselves from other people in the house until 10 days after their exposure to a close contact. - Persons with symptoms of COVID-19 can leave home and continue with normal activities if they have tested negative at least once since being exposed. - Persons with no symptoms of COVID-19 can leave home and continue with normal activities, so long as they remain symptom free. |
| **A person who tests positive to COVID-19 but doesn’t develops symptoms should*:*** | - Stay at home for at least 2 days and then return to activities since they do not have symptoms. - *Stay home for at least 10 days after the date of their positive test result.* - Stay at home for at least 14 days after the date of their positive test result. - Feel free to go out because they do not feel sick. |
| **Which of the following most accurately describes the City of San Francisco’s guidance for COVID-19 testing?** | - Only those people who have been exposed to someone with COVID-19 within the past 14 days can get tested. - Only those people who have symptoms of COVID-19 within the past 10 days can get tested. - *Anyone can get tested if they either: have one symptom, or they have been exposed to someone with COVID-19 within the past 14 days, or they are a frontline worker, regardless of symptoms or exposure.* - Only frontline workers can get tested. |
| **What information is NOT collected during the Contact Tracing Interview?** | - Demographic information about the contact. - The contact’s living situation. - *The contact’s annual income.* - The contact’s current symptoms. |
| **Which of the following City Test sites have a set number of reserved appointments exclusively for contacts reached by the SFDPH Contact Tracing Program?** | - *Embarcadero Test Site* - Castro Mission - Alternative Testing Site - City College Test Site - SOMA Test Site |
| **Which of the following describes the procedures that contact tracers should use to make food referrals for close contacts during their isolation or quarantine period?** | - Send an email to Amazon Fresh with preferred food options - Call the I&Q Feeding Unit and inform them you want to make a food referral. - *Document the referral in the CommCare Contact Monitoring Form.* - All of the above. |
| **Which of the following is NOT offered by the I&Q Unit to support isolation and quarantine?** | - Food - Cleaning supplies - *Childcare* - Isolation hotel rooms |
| **Who is eligible to receive food support from the I&Q Unit?** | - Anyone who does not have enough food to last for the duration of their isolation or quarantine period, but no other members of their household. - Close contacts who are already receiving food benefits from the city but want food for their extended family in case they need to quarantine too. - *Any close contact who does not have enough food to last for the duration of their isolation or quarantine period, and other members of their household.* - Any low-income resident who is required to isolate or quarantine due to COVID. |
| **While logged in to Amazon Connect, a contact tracer who does not want to accept calls so that they can work on contact monitoring notes or make food/cleaning referrals after completing a contact tracing interview, should set their Amazon Connect status to:** | - Online - Offline - *Break* - Available |
| **True or false: contact tracers can send AND receive messages from contacts using Amazon Connect.** | - True - False |
| **Which of the following most accurately describes some of the primary activities performed in Microsoft Teams by the SFDPH Contact Tracing Program?** | - Making outgoing calls and sending messages to contacts and cases. - Sharing documents, having in-brief and out-brief meetings, and messaging with other members of the contact tracing team. - Writing and saving clinical notes for case and contact files. - Entering, saving, and managing data during the contact tracing interview. |
| **Using CommCare, which steps must you follow to access information about the contacts that have been assigned to you at the beginning of each shift?** | - Log-in, sync, check-in, then open the Unassigned Open Contacts folder. - Log-in, check-in, sync, open the All Contacts folder and look for contacts with your names assigned to them. - *Log-in, sync, check-in, then open the CommCare inbox and look for a “SECURE” email from your Team Lead.* - Log-in, sync, check-in, then open the My Contacts folder. |
| **Using CommCare, which of the following most accurately describes how contact tracers will know which of their assigned contacts should be prioritized?** | - In the My Contact list, enter “high-risk” in the search bar. - In the My Contact list, enter “symptomatic”, “age”, or “comorbidity” in the search bar. - *Sort the list by “Priority” and/or look for the red arrow shapes, which indicate a high priority contact.* - Sort the list by “Symptoms” – contacts with symptoms will be elevated to the top of the list. |

*Correct answers in italics.

| **Level 2. Post-training Self-efficacy survey** | |
| --- | --- |
| **How confident are you that you will be able to make cleaning and food support referrals for your clients using a standardized email template and an online supply ordering website?** | Very confident, Fairly confident, Somewhat confident, Slightly confident, Not at all confident |
| **How confident are you that you will be able to navigate a large manual of standard operating procedures to find specific guidance on how to do various activities of a contact tracer?** | Very confident, Fairly confident, Somewhat confident, Slightly confident, Not at all confident |
| **How confident are you that you will be able to schedule testing appointments for a client or make recommendation about where to get tested using the SF.Gov Testing Website ?** | Very confident, Fairly confident, Somewhat confident, Slightly confident, Not at all confident |
| **How confident are you that you will be able to use the CommCare COVID19 Tracking app to search, enter, and save data during contact tracing interviews?** | Very confident, Fairly confident, Somewhat confident, Slightly confident, Not at all confident |
| **How confident are you that you will be able to use the Amazon connect soft-phone app to make calls to your assigned contacts and other members of your contact tracing team?** | Very confident, Fairly confident, Somewhat confident, Slightly confident, Not at all confident |
| **How confident are you that you will be able to attend meetings, access files, and chat with other members of your contact tracing team during shifts using the Microsoft Teams app?** | Very confident, Fairly confident, Somewhat confident, Slightly confident, Not at all confident |
| **How confident are you that you will be able to use several applications at the same time (CommCare, Amazon Connect, and Teams) while simultaneously conducting an effective contact tracing interview (using health coaching, maintaining confidentiality, building rapport, minimizing implicit biases, etc.)?** | Very confident, Fairly confident, Somewhat confident, Slightly confident, Not at all confident |

**Level 3. Performance-based Evaluation rubric**

**COVID 19 Contact Tracing Call Quality Assurance Checklist**

**Name of Contact Tracer (CT):**

**Name of Shadowing Contact Tracer:**

**Date of Call:**

**Duration of Call:**

| **Introduction** | **YES** | **PARTIAL** | **NO** | **N/A** |
| --- | --- | --- | --- | --- |
| - Did the CT identify themselves with the health department? |  |  |  |  |
| - Did the CT discuss the purpose of the call/ask permission to talk about COVID-19? |  |  |  |  |
| - Did the CT verify identity of the contact (DOB, age, etc.)? |  |  |  |  |
| - Did the CT state all information collected will be confidential? |  |  |  |  |
| - Did the CT inquire about preferred language for communication? |  |  |  |  |
| - Did the CT collect/verify all the relevant demographic information? (beginning & end of call) |  |  |  |  |
| Comments: | | | | |
| **Symptoms / Medical Conditions** | **YES** | **PARTIAL** | **NO** | **N/A** |
| - Did the CT assess symptoms? |  |  |  |  |
| - Did the CT document if the contact was immunocompromised? |  |  |  |  |
| - Did the CT document if the contact was pregnant? |  |  |  |  |
| - Did the CT assess existing medical conditions? |  |  |  |  |
| Comments: | | | | |
| **Exposure** | **YES** | **PARTIAL** | **NO** | **N/A** |
| - Did the CT assess the contact’s living and housing situation? |  |  |  |  |
| Comments: | | | | |
| **Recommendations and Follow up** | **YES** | **PARTIAL** | **NO** | **N/A** |
| - Did the CT assess the contact’s ability to quarantine / isolate? |  |  |  |  |
| - Did the CT correctly categorize the contact risk? |  |  |  |  |
| - Did CT continue over to PUI form (if applicable)? |  |  |  |  |
| Comments: | | | | |
| **Technical Review** | **YES** | **PARTIAL** | **NO** | **N/A** |
| - Did the CT leave a complete encounter note? |  |  |  |  |
| - Did the CT successfully leave a voice mail/send text message (if applicable)? |  |  |  |  |
| - Did the CT navigate their way through standard operating documents (if needed)? |  |  |  |  |
| - Did the CT successfully use interpreter services (if needed)? |  |  |  |  |
| Comments: | | | | |

| **Referrals** | **YES** | **PARTIAL** | **NO** | **N/A** |
| --- | --- | --- | --- | --- |
| - Did the CT set up an appointment for testing (if applicable)? |  |  |  |  |
| - Did the CT explain the need to get a second test at the end of quarantine (if first test is negative)? |  |  |  |  |
| - Did the CT successfully refer contact to I&Q services? |  |  |  |  |
| Comments: | | | | |
| **Client-centered practice/motivational interviewing** | **YES** | **PARTIAL** | **NO** | **N/A** |
| - Did the CT ask open-ended questions to assess knowledge? |  |  |  |  |
| - Did the CT use demonstrate listening through reflective statements (using contact’s own words, repeating back information stated by the contact)? |  |  |  |  |
| - Did the CT provide accurate information? |  |  |  |  |
| - Did the CT ask contacts to restate new information in their own words? |  |  |  |  |
| - Did the CT state, “I don’t know but I will find out and get back to you” when asked questions they did not have the answer to? |  |  |  |  |
| - Did the CT addressing any challenging situations with the patient’s best interest and safety in mind? |  |  |  |  |
| - Did the CT address all of the contact’s questions or concerns and ask for any additional ones? |  |  |  |  |
| - Did the CT use comments & tone that were friendly and not judgmental? |  |  |  |  |
| - Did the CT have a natural flow to the interview? |  |  |  |  |
| - Did the CT close the call in a caring and reassuring manner? |  |  |  |  |
| Comments: |  |  |  |  |

**2 Supplementary Training Curriculum Data**


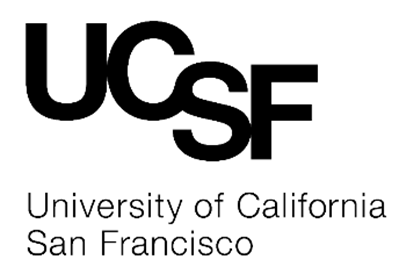

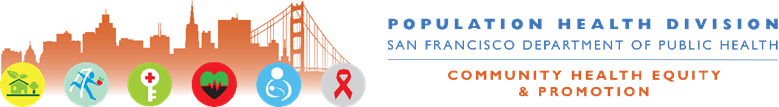
Contact Tracing Training

**Training Agenda**

**Training Agenda* for SFDPH Contact Tracing Training**

September 28-October 2

**Note: All trainees attending this training for San Francisco contact tracers must have completed the California Virtual Training Academy (VTA) Contact Tracing course. A certificate of completion of the VTA course will be required as soon as you receive it.**

**All materials can be found in shared folder**

**Office hours: schedule for technical support with CommCare, Amazon Connect, and Microsoft Teams.**

| **Day 1: Monday: SELF-STUDY & Mandatory CHECK-IN with Alisa and Anika (drop in 11-12pm)** | | | | |
| --- | --- | --- | --- | --- |
| To be Completed Before the Day 2 Live Web Meeting **(1 hour 15 minutes total):**   1. Complete the Pre-training Survey **(15 minutes)** 2. **For Module 1:** Overview of the SFDPH COVID-19 Response (**30 minutes**)    - Briefly read through the *COVID-19 Standard Operating Procedures for Case Investigation & Contact Tracing.pdf*. Focus on sections related to “Contact Tracing”.    - Read the SFDPH’s *Home Isolation & Quarantine Guidelines and Health Directive.*    - Visit the SF.Gov Get Tested website and: a) use the map search function to locate the testing site that is nearest to your place of residence; b) locate any testing site (s) where you would qualify to get tested; c) identify the current waiting time for a test at any of the sites you listed in b. 3. **For Module 2:** Making Calls with the Amazon Connect Softphone **(15 minutes)**    - You will receive your Amazon Connect login credentials. Login using a web browser (Chrome works best). 4. **For Module 3:** Contact Tracing Team Meetings and Communications with Microsoft Teams (**15 minutes)**    - Watch the Microsoft Teams short demonstration video.    - Watch Microsoft Teams video lesson. 5. Optional: watch Dr. Fauci and Mayor Breed talk about SF Contact Tracing and Case Investigation! | | | | |
| **Day 2: Tuesday LIVE ZOOM MEETING** | | | | |
| **Day** | **Time** | **Module Name** | **Required materials** | **Facilitator (s)** |
| 2 | 9:00-9:15  (15 min) | **Training Introduction** | - Training Intro PowerPoint Presentation slides  - SF Epidemiology | Alisa Jenny and Anika Kalra, UCSF |
| 2 | 9:15-9:30  (15 min) | **Module 1:** Introduction to the SOP Manual | - Mod. 1 PowerPoint Presentation slides | *See facilitators below* |
| 2 | 9:30-10:00  (30 min) | Skill Development Activity 1: *Join a CT SOP Scavenger Hunt* | - SOP for Case Investigation & Contact Tracing  - Skill Development Activity Packet Activity 1 | Anika Kalra, UCSF |
| 2 | 10:00-10:15  (15 min) | ***1b****.* Making Referrals to the Isolation and Quarantine Unit | - Mod. 1 PowerPoint Presentation slides  - Email template from SOP | Alisa Jenny, UCSF |
| 2 | 10:15-10:30  (15 min) | **1c.** Making COVID-19 Test Appointments for Contacts. | - Mod. 1 PowerPoint Presentation slides  - SF.Gov Get Tested website | Anika Kalra, UCSF |
|  | 10:30-10:45  (15 min) | **Break** |  |  |
| 2 | 10:45-11:15  (30 min) | **Module 2:** Making Calls with the Amazon Connect Softphone | - Mod. 2 PowerPoint Presentation slides  - Demo | UCSF Facilitator* |
| 2 | 11:15-11:45  (30 min) | Skill Development Activity 2: *Practice Making Calls with Amazon Connect* | - Web-browser, open to: Amazon Connect  - Your Amazon Connect login username and password  - Skill Development Activity Packet Activity 3 | UCSF Facilitator* |
| 2 | 11:45-12:00  (15 Min) | **Module 3:** Contact Tracing Teams Meetings and Communications with Microsoft Teams | - Mod. 3 PowerPoint Presentation slides | SFDPH Facilitator* |
| 2 | 12:00-12:30  (30 min) | Skill Development Activity 3: *Practicing Teams* | - Teams Scavenger Hunt | SFDPH Facilitator* |
| 2 | 12:30-1:00  (30 min) | **Break** |  |  |
| 2 | 1:00-1:45  (45 min) | Panel Discussion | - Panel discussion with expert CTs |  |
| 2 | 1:45-2:00  (15 min) | Recap and Closing | - Reminder to do your homework | Alisa or Anika, UCSF |

| **Day 3: Wednesday morning: SELF-STUDY & OFFICE HOURS** | | | | |
| --- | --- | --- | --- | --- |
| To be Completed Before the Day 3 Live Web Meeting **(1 hour 15 minutes total):**   1. **For Module 4**: Data Entry and Management with the CommCare COVID-19 Tracking App **(30 minutes)**    1. You will be emailed a CommCare demo environment username and log-in in the invitation for training    2. Log-in to the CommCare demo environment and explore it. 2. **For Module 5: Putting it All Together (30 minutes)**    1. Practice reading the Script with a friend or family member acting as the contact.    2. View video demonstration of using CommCare (you will be doing this tomorrow) **^x^**       1. **^x^**video mentions testing, but does not show how to make a testing referral 3. **For Amazon Connect:** walk through slides and practice.   **Drop-in office hours for CommCare, Microsoft Teams, and Amazon Connect IT support –** *OPTIONAL, if you cannot access or log-in to any of these apps –* Schedule. | | | | |
| **Wednesday afternoon - LIVE ZOOM MEETING** | | | | |
| **Day** | **Time** | **Module Name** | **Required materials** | **Facilitator (s)** |
| 4 | 12:00-12:15  (15 min) | **Welcome** | - Welcome/put something in the chat  - What we went over yesterday  - What today looks like  - Mention technology | Alisa Jenny, UCSF and Anika Kalra, UCS |
| 4 | 12:15-12:30  (20 min) | **Module 4**: Data Entry and Management with the CommCare COVID-19 Tracking App | - Mod. 2 PowerPoint Presentation slides | Alli Foreman, UCSF |
| 4 | 12:30-1:30  (60 min) | Skill Development Activity 4: *Practice viewing, searching, and entering data in CommCare* | - Web-browser, open to: CommCare Demo Environment  - Your CommCare demo login username and password  - Skill Development Activity Packet Activity 5 | Alli Foreman, UCSF |
|  | 1:30-1:45  (15 min) | **Break** |  |  |
| 4 | 1:45-2:00  (15 min) | **Module 5:** Putting it All Together | - Mod. 5 PowerPoint Presentation slides | Shayanne Martin, UCSF |
| 4 | 2:00-4:00  (2hr) | Skill Development Activity 5: *Conduct a Contact Tracing Interview while Entering Data in CommCare.* | - Web-browser, open to: CommCare demo environment.  - Your CommCare login username and password  - Skill Development Activity Packet Activity 6  - Group Discussion + Debrief | Shayanne Martin, UCSF |
| 4 | 4:00-4:15  (15 min) | **Close of Training:** Q&A, Next Steps and Closing Remarks | - Shadow and onboarding shifts schedule. | Anika Kalra, UCSF |

| **Day 4: Thursday SELF STUDY & TESTS**  To be completed after Day 3 of the course:   1. **For Module 5: review CommCare Demo environment**     1. Watch video on Person Under Investigation (PUI) referrals 2. **CommCare TEST** to be completed by midnight on Sunday – must get a 70% score or higher to continue on to training and onboarding shifts **(1 hour).** 3. **Knowledge TEST** to be completed by midnight on Sunday – must get a 70% score or higher to continue on to training and onboarding shifts **(1 hour)** |
| --- |

| **Next 2 weeks: Training and Onboarding shifts^+^**  **^+^Before training shift: go to office hours to confirm that you can login & use CommCare live and Amazon Connect – view schedule.**  **Training shift schedule:**   1. Wednesday 8:30-11:30am or Friday 8:30-11:30am   **Onboarding shift schedule:**   1. Monday – Friday (everyday) 12-4pm |
| --- |

*Facilitator names and file links have been removed for privacy. Additional materials are available upon reasonable request to corresponding author.
